# Determining the Effects of Inter-Layer Time Interval in Powder-Fed Laser-Directed Energy Deposition on the Microstructure of Inconel 718 via In Situ Thermal Monitoring

**DOI:** 10.3390/ma17030538

**Published:** 2024-01-23

**Authors:** Evan Handler, Aref Yadollahi, Yucheng Liu, Scott M. Thompson

**Affiliations:** 1 Department of Mechanical Engineering, Mississippi State University, Mississippi State, MS 39762, USA; 2 Department of Mechanical & Civil Engineering, Purdue University Northwest, Hammond, IN 46323, USA; arefy@pnw.edu; 3 Department of Mechanical Engineering, South Dakota State University, Brookings, SD 57007, USA; yucheng.liu@sdstate.edu; 4 Department of Mechanical & Aerospace Engineering, University of Missouri, Columbia, MO 65211, USA

**Keywords:** additive manufacturing, laser engineered net shaping, thermal monitoring, quality control, microstructure, melt pool

## Abstract

Cylindrical Inconel 718 specimens were fabricated via a blown-powder, laser-directed energy deposition (DED-L) additive manufacturing (AM) process equipped with a dual thermal monitoring system to learn key process–structure relationships. Thermographic inspection of the heat affected zone (HAZ) and melt pool was performed with different layer-to-layer time intervals of ~0 s, 5 s, and 10 s, using an infrared camera and dual-wavelength pyrometer, respectively. Maximum melt pool temperatures were found to increase with layer number within a substrate affected zone (SAZ), and then asymptotically decrease. As the layer-to-layer time interval increased the HAZ temperature responses became more repetitive, indicating a desirable approach for achieving a more homogeneous microstructure along the height of a part. Microstructural variations in grain size and the coexistence of specific precipitate phases and Laves phases persisted among the investigated samples despite the employed standard heat treatment. This indicates that the effectiveness of any post DED-L heat treatment depends significantly on the initial, as-printed microstructure. Overall, this study demonstrates the importance of part size, part number per build, and time intervals on DED-L process parameter selection and post-process heat treatments for achieving better quality control.

## 1. Introduction

Additive manufacturing (AM) is a means for producing physical 3D objects layer-by-layer directly from computer-aided design (CAD) models using a variety of materials [1]. Compared to traditional manufacturing technologies such as machining, casting, forming, etc., AM provides a means to fabricate complex, customized parts from the ground-up. During the past three decades, AM has evolved significantly, resulting in the popular metals AM methods such as power bed fusion (PBF) and directed energy deposition (DED) [2]. 

DED uses a high-intensity heat source, such as a laser or electron beam, to melt powder- or wire-fed metal in a user-generated pattern layer-by-layer until the final part is achieved. Laser Engineered Net Shaping^®^ (LENS) is a commercial, blown-powder laser DED process (DED-L). The LENS process, along with other DED-L processes, offers some advantages over PBF methods such as less obstructed process inspection, including thermal inspection of exposed surfaces during the build [3,4,5,6], and their ability to repair components and create functionally graded materials (FGMs) [7,8]. 

Due to its thermal capacitance, the spatiotemporal temperature distribution of a part during its DED-L will exist and depend on process planning (time intervals between successive layer deposits at the same location), process parameters (travel speeds, laser power), substrate type/size, material type, material size, part number, and more. The microstructural evolution and eventual mechanical properties of metal DED-L parts depend on this transient temperature distribution, i.e., thermal history. Yadollahi et al. [9] found that longer inter-layer time intervals result in higher local cooling rates, thus leading to finer microstructures, higher tensile strength, and lower elongation-to-failure in DED-L 316 stainless steel samples. Other studies also show that the microstructural evolution of a metal DED-L part can be altered significantly by changing inter-layer time intervals [10,11,12,13]. A common observation is that the time dedicated to the dissipation of accumulated heat has a direct impact on microstructure and its phases.

For a DED-L process with constant inter-layer time intervals, Zheng et al. [13] found that bulk heating can occur in smaller specimens, therefore causing layer-dependent cooling rates, resulting in the average part temperature increasing steadily with the number of layers. They also demonstrated that, as the inter-layer time interval decreases, by decreasing the idle time of the deposition head (i.e., the time during which the deposition head does not move), the severity of bulk heating increases significantly and that the initial temperature of the previously deposited layer will affect the cooling/solidification rates of the subsequent layer [13,14]. Previous work has also identified a ‘substrate affected zone’ (SAZ) of a part during DED [15]. This SAZ is where heat transfer to the substrate is more significant, and a heatsink effect is evident. 

Nickel-based superalloys are widely used in applications requiring sustained material performance in harsh environments at elevated temperatures, such as gas turbines, nuclear reactors, and aerospace components. Inconel 718 is an austenitic, precipitation-hardening nickel–chromium–iron superalloy known for its high strength and resistance to oxidation at high temperatures [16]. As it is difficult to machine, expensive, yet weldable, it has become a good candidate for AM [17]. Similar to wrought materials, the typical microstructure of AM Inconel 718 consists of different phases, including: the face-centered cubic (fcc) γ phase composed of Ni-Cr-Fe, which is the matrix phase; the fcc γ′ phase composed of Ni_3_Al/Ti, which is a strengthening precipitate coherent with the matrix; the body centered tetragonal (bct) γ″ phase composed of Ni_3_Nb, which is the predominant strengthening precipitation in this alloy; the orthorhombic Ni_3_Nb δ-phase, which is a non-hardening precipitate; and the Nb-rich Laves phase and metal-carbide (MC) particles [18].

Many have studied the blown-powder DED-L of Inconel 718 specimens with respect to their process–structure–property relationships. Zhang et al. optimized parameters for controlled solidification and target microstructures [19] and Shah et al. demonstrated DED-L’s ability to tailor mechanical properties and selectively control hardness/wear resistance through process parameters and carbide formation [20,21]. Corbin et al. identified laser power and working distance as primary influencers of bead/track dimensions and hardness [22], while Segerstark et al. linked thermal history (via process parameters) to grain structure, phase transformation, and cracking susceptibility [23]. Kuriya et al. established a link between solidification time and porosity, suggesting porosity control through optimized solidification time [24].

The mechanical properties of AM parts depend strongly on local microstructures and defects which all depend on the use of optimal process/design parameters. Hence, it is ideal to monitor AM processes to better ensure product quality and process efficiency. This is especially true for the AM of high-value components for critical applications. Data obtained by in situ monitoring of the build process can be used to more efficiently adjust process parameters to gain more control over a part’s thermal history and solidification rates/directions. Monitoring data may also be used for part qualification and retained for product warranties. There are several methods for monitoring the DED-L process, with thermal monitoring being the most popular since it can be directly related to local heat transfer and metallurgical bonding. Thermal monitoring of the DED-L process is commonly achieved using infrared (IR) cameras and/or dual wavelength (DW) pyrometers to measure surface temperature fields (and heat transfer) and absolute melt pool temperatures, respectively [4,25,26,27,28,29,30,31,32,33]. Through thermal monitoring, various process metrics such as local surface temperature, cooling/solidification rates, and thermal gradients can be directly or indirectly obtained and used for predicting the presence of defects, the resultant microstructure, and the mechanical properties of DED-L parts [4]. In addition, thermal monitoring methods have been used to achieve feedback control loops for adaptive laser power control to maintain a constant melt pool temperature/size or even ‘feed-forward’ loops for predicting heat transfer at later points during manufacture and offsetting process parameters accordingly [34,35,36]. Marshall et al. [4] combined DW pyrometry and IR camera monitoring (dual thermography) for layer-wise melt pool calibration and analysis in Ti-6Al-4V DED-L. Melt pool geometries, maximum temperatures, and cooling rates were estimated and solidification maps were used in conjunction to better understand microstructure formation. Gibson et al. [37] used in-axis IR thermography for real-time melt pool characterization in large-scale DED-L, enabling closed-loop quality control. Khanzadeh et al. [38,39] monitored melt pool temperatures and linked them to porosity via self-organizing maps and X-ray tomography, achieving 96% success rate in porosity detection.

Although process parameters can be altered to make a part’s thermal history more isothermal with time, post-AM heat treatments may still be needed to fully or partially rectify inhomogeneous microstructures due to a non-uniform thermal history [40]. A wide range of heat treatments are commonly used for Inconel 718. In addition to temperature, various solution treating and aging time intervals can be employed to generate desired microstructures and mechanical behaviors [41,42]. In its as-built condition, AM Inconel 718 lacks the γ″ and γ′ strengthening precipitates required to achieve nominal yield and ultimate strengths; however, with appropriate post-fabrication heat treatments, AM Inconel 718 can achieve strength properties commensurate with wrought product forms through precipitation of fine γ′ and γ″ strengthening phases and needle-like δ phases [41,42,43,44]. Relative to its wrought counterparts, AM Inconel 718 may benefit from slightly different heat treatment schedules (i.e., temperature and time intervals) to achieve the desired mechanical properties [45]. Qi et al. [46] studied and compared microstructures and tensile properties of DED-L Inconel 718 under as-deposited and heat treatment conditions. Heat treatments included: direct aging, solution treated and aging (STA), and full homogenization followed by STA. The results showed that the direct aging heat treatment produced the highest tensile strength while the homogenized STA produced the best ductility [46]. It can be concluded from these studies that the preeminent resultant microstructure (i.e., texture, porosity, etc.) and mechanical properties (both monotonic and cyclic) for DED-L Inconel 718 can be achieved through the appropriate selection of process parameters and post-process heat treatment.

Despite the beneficial use of thermal monitoring for DED process control and quality assurance, very little research has been conducted and reported on its application to the powder-fed DED-L of Inconel 718. In addition, the melt pool behavior and bulk thermal response of cylindrical parts has not been reported—and this must be considered when applying thermal monitoring to the DED-L of real-life components (as opposed to thin-walled structures). This study uses dual thermography to reveal the influence of time intervals on the resultant melt pool behavior, temperature fields, and microstructure of cylindrical DED-L Inconel 718 samples.

## 2. Materials and Methods

### 2.1. Additive Manufacturing Parameters

Vertical cylindrical rods of Inconel 718 were fabricated using an OPTOMEC LENS^®^ 750 system (with a 1 kW Nd:YAG laser). Each rod was produced individually on an unheated substrate of Inconel 718 with dimensions of 76.2 mm × 76.2 mm × 3.2 mm set atop a copper spacer measuring 6.35 mm in thickness. Rods were designed to possess a height of 76.2 mm and a diameter of 6.35 mm. Spherical Inconel 718 powder (Phelly Materials, Inc., Upper Saddle River, NJ, USA) prepared using a plasma rotating electrode process (PREP) with a mesh size of −100/+325 (d50: 230 µm, d90: 120 µm) was used in its as-received condition. The build chamber was sealed and purged with industrial grade argon for all builds. 

Process parameters, such as laser power, scan speed, and powder flow rate, were tested systematically, via trial-and-error inspection, to obtain parts of high dimensional accuracy and minimal porosity. Dimensional accuracy was tested by manufacturing test cylinders with nominal dimensions of 6.35 mm in diameter and 6.35 mm in height. Porosity was investigated by sectioning test cylinders and visually inspecting them using an optical microscope. The parameters that passed these inspections were rechecked by manufacturing a cylinder that measured 6.35 mm in diameter by 12.7 mm in height. The final process parameters used are summarized in Table 1. These process parameters were held constant for each layer during the manufacturing process. A rotating hatch pattern was used in intervals of 0°, 90°, 180°, and 270° to fill each layer. The cylindrical samples were built one-at-a-time using three different inter-layer/idle time intervals, i.e., 0 s, 5 s, and 10 s; herein referred to as the no-time-interval (NTI), low-time-interval (LTI), and high-time-interval (HTI) samples, respectively. Only the time interval was varied for each experiment; specimen geometry, DED-L process parameters, and scan strategies were all held constant. This was accomplished by imposing an idle time in the digital motion controller (DMC) code that was executed after the laser was turned off on the last hatch fill.

### 2.2. Thermal Monitoring

**Figure 1:**
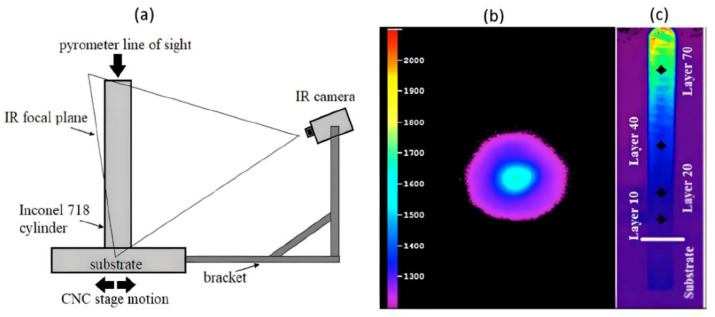
(**a**) Schematic showing IR camera (along with its focal plane) and pyrometer setup in relation to substrate and Inconel 718 cylinder within the powder-blow DED-L chamber (IR camera and substrate move together while pyrometer constantly stays above) as well as sample images of (**b**) the pyrometer and (**c**) the IR camera with the extracted points and substrate marked and labeled. HAZ is indicated by rising temperatures.

To quantify the heat transfer and temperatures of the process, as well as relate the microstructure of the DED-L Inconel 718 samples to process parameters, the AM of one specimen from each group was monitored using a DW pyrometer and IR camera. The IR camera (Sierra-Olympic) employed an uncooled microbolometer detector and was mounted to the CNC build stage within the OPTOMEC LENS chamber while the pyrometer was located atop the lens chamber and was aimed through the deposition head column (via a series of turning mirrors) to monitor the melt pool. Data collection rates for each instrument were not entirely constant and were approximated from timestamps in the raw data. The frame rates were found to be ~6.6 Hz for the pyrometer and ~6.4 Hz for the IR camera. The IR camera was blackbody temperature calibrated, and a material-specific calibration was also performed using the methods reported by Marshall et al. [4]. This calibration approach consisted of comparing IR-measured temperatures within the melt pool region during the contour deposit of a new layer (when the melt pool intersected the IR camera focal plane) with pyrometer-measured temperatures recorded at a similar time. This approach generated a linear relationship used for offsetting raw temperature measurements from the IR camera. In this study, it is assumed that the calibrated IR temperature measurements (IR images) are only an approximation of the true, absolute temperature field existing along the exposed portion of the part. Errors associated with the IR-measured temperatures exists due to employing (as input to the IR camera) a constant, phase- and temperature-independent emissivity of Inconel 718. For instance, Greene et al. demonstrated that the hemispherical emissivity of solid Inconel 718 ranges from 0.20 at 200 °C to 0.33 at 1000 °C [47]. Figure 1a illustrates the setup of the thermal cameras and their position with respect to the build. The starting position of the deposition head was set so that it would intersect the middle of the IR camera’s field of view during manufacture. Calibrated data were imported into MATLAB^®^ (Version 8.5 (R2015a)) for analysis. Figure 1b,c show representative sample images from the DW pyrometer and IR camera, respectively. Note that the layers of interest include the 10th, 20th, 40th, and 70th layers herein referred to as L10, L20, L40, and L70, respectively.

### 2.3. Microstructure Evaluation

To study the effect of process time intervals and post-AM heat treatment on microstructural characteristics of the Inconel 718 specimens, the fabricated cylindrical samples were sectioned into thirds—each having a length of ~12 mm. The lower (near the substrate) and middle sections, near L20 and L50, respectively, were investigated in detail for this study. The selected specimens underwent a standard heat treatment for Inconel 718, per AMS 5664 E [48], consisting of solution treating at 925 °C for half an hour followed by air cooling; aging at 725 °C for 8 h, then furnace cooling to 625 °C and treating for a total of 10 h, followed by air cooling. This heat treatment is a type of solution treatment, which involves heating an alloy to a suitable temperature and holding it at that temperature long enough to cause one or more constituents (precipitates) to enter into a solid solution and then cooling it rapidly enough to hold these constituents in the solution [48]. After the solution treatment, these samples were hot mounted in PolyFast and then ground and polished before being etched using Kallings etchant for about 10 s. Imaging was performed on an optical microscope (ZEISS Axiovert 200, Jena, Germany).

## 3. Results and Discussion

### 3.1. Melt Pool Behavior

**Figure 2:**
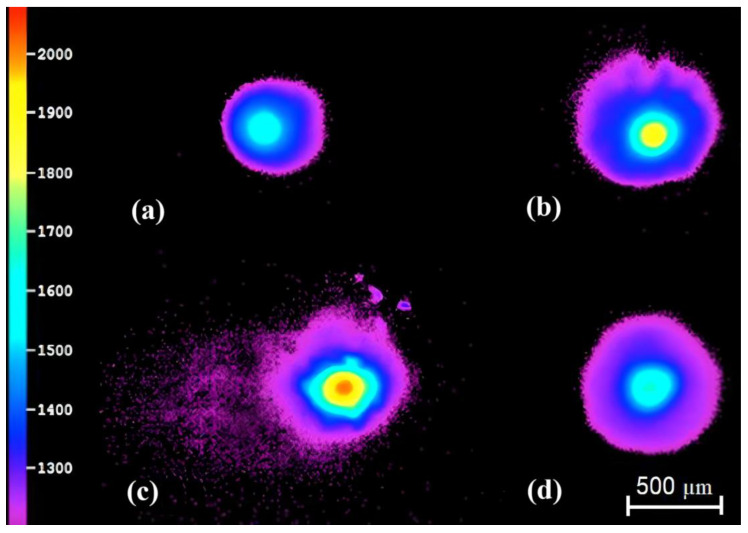
Pyrometer images of melt pool during the DED-L of Inconel 718 during the NTI condition at different layers: (**a**) L1, (**b**) L5, (**c**) L10, (**d**) L20. The temperature scale is Celsius. Size scale bar located at bottom right.

Aerial melt pool thermal images were obtained via DW pyrometry during the blown-powder DED-L of Inconel 718 cylinders (one at a time) for the NTI, LTI, and HTI process conditions. Representative images of the melt pool obtained during the NTI condition for layers L1, L5, L10, are L20 are shown in Figure 2. The images were collected approximately halfway through layer fabrication for each inspected layer. The orientation of the melt pool varies in each image due to the circular contour and cross-hatching fill pattern used to generate each layer.

From Figure 2, it can be seen that the lowest observable temperature of the melt pool is ~1200 °C, occurring along its outer, mushy zone region which is where solid- and liquid-phase Inconel 718 alloy co-exist (melting temperature range for Inconel 718 is 1260–1336 °C). All recorded melt pools are semicircular in nature due to the use of a Nd:YaG laser with a Gaussian intensity profile during DED-L. Layer-wise variation in the melt pool temperature distribution and its maximum temperature (at the center of each melt pool) is apparent when observing the melt pools shown in Figure 2. The maximum melt pool temperature increases when going from L1 to L5 and then to L10, but then it decreases during L20. In fact, the melt pool temperature measured for L20 was found to be cooler than that for L1 (as evidenced by more purple). 

For L1, the melt pool is smaller in size than for L20, and this is attributed to the unique heat transfer occurring at layers near the substrate. During the deposition of L1, the laser heat flux travels along a smaller conduction path and the substrate allows the heat flux to diffuse and dissipate more rapidly. The L5 melt pool, which is slightly hotter than that of L1, achieves temperatures around 1850 °C in the region directly under the laser. Such elevated temperatures are ≳500 °C above the liquidus of Inconel 718 indicating a high level of superheat in the melt pool. The L10 melt pool achieves temperatures of ~2000 °C which is ~8% higher than that of L5. 

As shown in Figure 2c, the L10 melt pool is the most unique in terms of features, including very high temperatures, evidence of spatter/ejecta, lower circularity, and ‘trailing saturation’, i.e., the region behind the melt pool with several dots present. This saturation is most likely due to the vaporization of, and plasma formation around, the melt pool and the very high temperature difference affecting pyrometer measurements. Spatter is observed near the advancing front of the melt pool and possess temperatures within/near the solidification temperatures of Inconel 718. These ejected fluid volumes from the melt pool exist due to very high vapor recoil pressures forming within the melt pool which leads to vapor expulsions and their carrying of fluid mass. These ejected droplets solidify into semi-circular and non-circular shapes mid-flight. The instability of the melt pool, as evidenced by its reduced circularity, is attributed to the high level of superheat observed. As the temperature difference between the top and bottom of the melt pool increases, more advection in the melt pool occurs due to natural and Marangoni convection. The increased melt pool advection can lead to a morphology that varies more with time. This indicates that when the part contains more initial heat (prior to depositing a new layer), the melt pool is more likely to be unstable and hotter. Circularity appears to decrease with maximum melt pool temperature—as evidenced by L5 and L10 in Figure 2.

**Figure 3:**
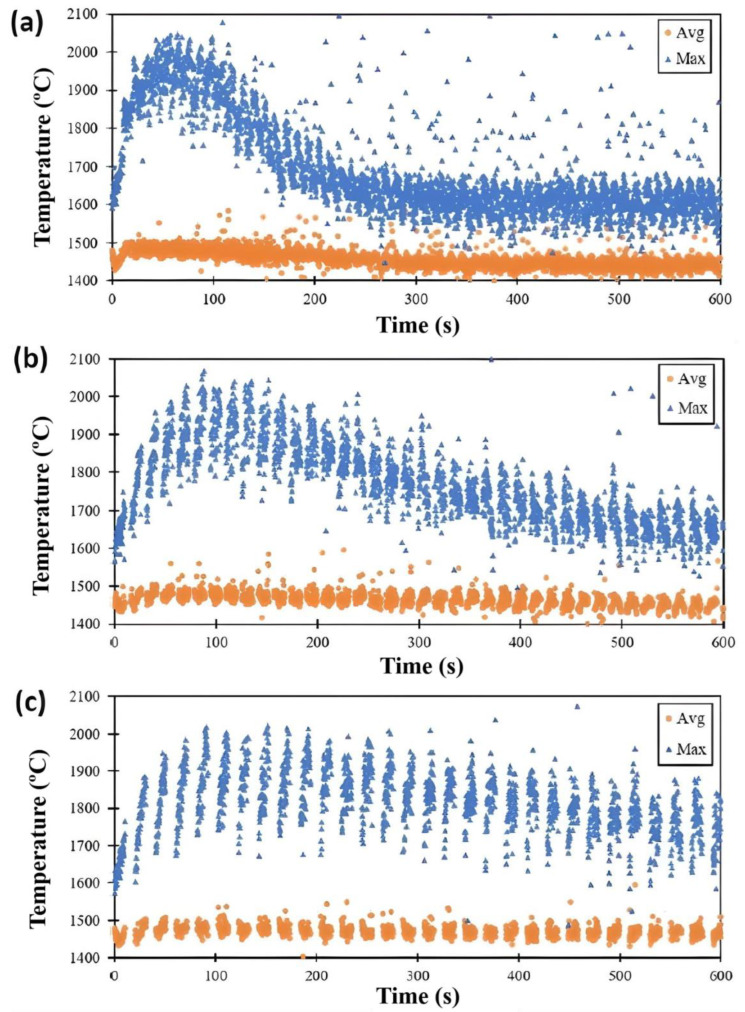
Average (“Avg”) and maximum (“Max”) melt pool temperatures of (**a**) NTI (0–300 s), (**b**) LTI (0–800 s), and (**c**) HTI (0–900 s) builds.

Figure 3 shows the average and maximum melt pool temperatures for the different time intervals investigated, i.e., NTI, LTI, and HTI. Average melt pool temperatures were calculated by taking the mean temperature of all temperatures greater than the liquidus temperature of Inconel 718 (1336 °C), while the maximum melt pool temperature was taken as the highest temperature recorded.

From Figure 3 it can be seen that the maximum temperature varies greatly during the deposition of a layer for all conditions investigated while the average melt pool temperature is relatively constant with respect to layer number. The sporadic jumps in maximum temperatures may be attributed to superheated melt pool instability which also impacts the reflected laser emission captured by the DW pyrometer. These spontaneous temperature jumps in the melt pool can also be possible if conditions allow for more superheating of the melt pool. The NTI building condition resulted in a higher frequency of sporadic maximum temperatures. As the time interval increased, the sporadicity of the maximum melt pool temperature decreased. The range of the maximum melt pool temperature for a given layer increases with time interval. For instance, for the NTI condition, the maximum melt pool temperature can increase over 100 °C during a layer, while for the HTI condition, the range is ~300 °C.

It can be seen from Figure 3 that temperature data appear in layer-wise clusters demonstrating track-wise temperature evolution. The maximum melt pool temperature clearly changes with deposition of each track and layer—especially during initial layers. Interestingly, the data suggest that the maximum temperature can either increase or decrease along the track. For the HTI process conditions, it is apparent that the maximum temperature of the melt pool increases with track deposition for a given layer. This is confirmed by seeing that each layer-specific cluster of temperatures has a positive slope; ‘leaning’ to the right with respect to time. Although this trend is clear for the HTI process conditions, it becomes less clear for the shorter time intervals, namely the LTI and NTI process conditions. For the NTI and LTI process conditions, the maximum melt pool temperature appears to increase with layer deposition time for layers prior to the global maximum; however, later in the process, the melt pool temperature decreases with track deposition. These trends can be attributed to the ability of the part to dissipate heat via conduction and through the environment prior to the initiation of a new layer.

The global maximum in melt pool temperature vs. time appears to be an indicator of when the part becomes a better heat dissipator. The heat transfer due to convection and radiation, which strongly depends on surface area, increases with build time until the part is sufficiently tall so that its HAZ no longer penetrates the substrate. There is a noticeable point during the build in which this occurs, i.e., the data shown in Figure 3. It is at this point of the build that the thermal resistance due to conduction becomes more dominant than that due to convection/radiation. This contributes to the observed inflection in maximum measured melt pool temperature. This inflection can delineate the height of a substrate-affected zone (SAZ). Past this point, environmental heat transfer becomes sufficient to start reducing the stored thermal energy in the part. As is shown in Figure 2, the maximum melt pool temperature increases with layer number until L10, then L20 shows a much cooler melt pool, and this agrees with the data provided in Figure 3a. The increase in maximum melt pool temperature with hotter initial layer temperature agrees with other experimental and numerical DED-L investigations [49]. Costa et al. show that the average temperature increases with layer number and then stabilizes after ~5–10 s and this agrees with the average melt pool data in Figure 3 [14].

### 3.2. Heat-Affected Zone

**Figure 4:**
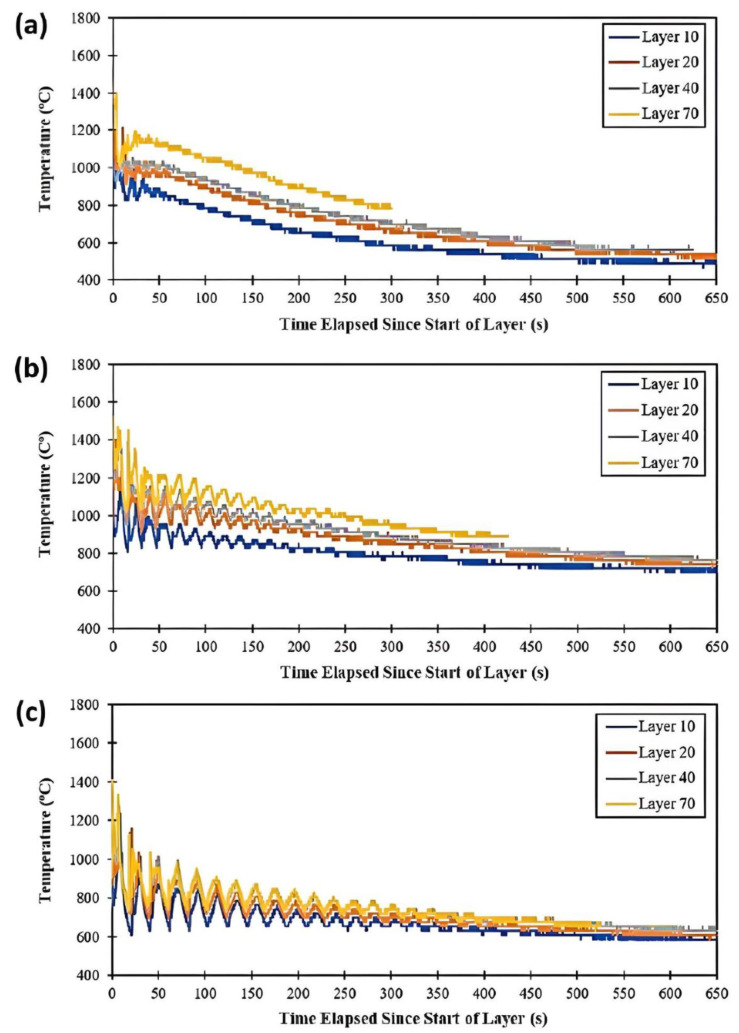
HAZ temperature responses/histories of different layers as measured via IR camera during (**a**) NTI, (**b**) LTI and (**c**) HTI process conditions.

Bulk temperature data gathered by the IR camera were extracted at predefined points of interest using a separate MATLAB code. The predefined points were selected within the IR camera focal plane at layers L10, L20, L40, and L70, corresponding to points closest to the camera, as shown in Figure 1c. The collected temperature data were plotted with respect to time to show different thermal histories. Figure 4a–c displays the bulk temperature data measured at different layers from the NTI, LTI, and HTI builds, respectively. Note that the origin for each curve shown in Figure 4 corresponds to the beginning of its respective layer fabrication. Therefore, the L70 thermal history has the shortest thermal history since the amount of fabrication above it was less than all other thermal histories. Data are presented in this manner so that the effects of build height on cooling rate are more easily discernable.

As expected, the results in Figure 4 show that the layer-to-layer time interval significantly affects the cooling time and introduces reheating as subsequent layers are deposited over the measured layer. The maximum temperature achieved for each measured layer’s thermal history was similar for all conditions investigated—being around 1400 °C, which is near the liquidus temperature of Inconel 718. These IR-camera-measured temperatures are representative of spatially averaged melt pool temperatures intersecting the IR camera focal plane during DED-L. The lowest and highest layers observed, L10 and L70, provided for a thermal history with lower and higher temperatures for all time intervals investigated, respectively. This demonstrates that the part retains heat during the DED-L process; however, temperature histories begin to coalesce as the layer-to-layer time interval increases, i.e., going from the NTI condition to the HTI process condition. Thus, using a high time interval during DED-L would more likely lead to a more uniform microstructure along the build height. The amplitude of temperature rises/drops during DED-L, or the degree of thermal cycling, increases with the time interval employed. The more severe temperature rises/drops shown for the HTI building condition can result in higher heating/cooling rates, thus providing conditions conducive to finer microstructures. As the time interval decreases, to the limiting NTI condition, the amplitude of the HAZ temperature cycles decreases more rapidly with each layer. This provides for lower cooling rates since bulk heating of the part is more prominent and the initial layer temperatures are more elevated (preheated).

### 3.3. Microstructure

**Figure 5:**
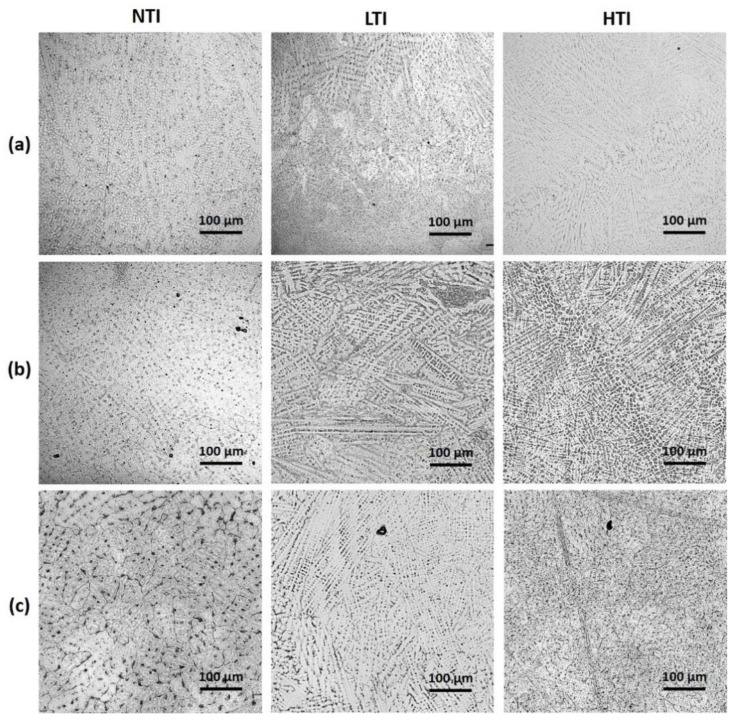
Microstructure images of (**a**) L20, (**b**) L50, and (**c**) heat-treated samples (all rows: left, NTI, center, LTI, right, HTI. All images are at 20× magnification).

Optical micrographs of DED-L Inconel 718 samples manufactured using the three different layer-to-layer time intervals (i.e., NTI, LTI, and HTI), are shown in Figure 5. Two sections were chosen for microstructural characterization: one near L20 and the other near L50. Analysis of the melt pool temperatures shows that all build conditions result in the rods undergoing SAZ-type heat transfer in which the initial layer temperature increases with height, followed by a peak/inflection in melt pool temperature, followed by an environmental cooling regime where the maximum melt pool temperature decreases with height until a steady state is achieved. This evolving heat transfer is evidenced in the L20 sample where a transitional microstructure is observed. Note that in all the time intervals investigated, the critical layer corresponding to maximum melt pool temperature inflection was below L20. The L20 microstructure is clearly different from what was observed for the L50 sample, where a less-evolutionary, steady-state track-wise temperature response was reached. For layers beyond L50, a similar microstructure was observed as that of the L50 section. Microstructural analysis revealed a mixture of equiaxed and dendritic structures near the edge of the specimens, which changes to entirely dendritic structures as the location moves away from the circumference towards the center of the analyzed section. Comparing the time-temperature-transformation (TTT) charts obtained for wrought Inconel 718 [50] to the plotted bulk temperature curves, as shown in Figure 5, material phases that will be formed in the builds can be predicted. As the initial cooling rates upon solidification of the melt pool are very high, they do not allow for much phase transformation, and the TTT chart shows that the primary phase should be the initial γ matrix with significant δ and some Laves phases mixed in. However, in the LTI and HTI builds, it is hard to observe these phases. This could be attributed to the lower reheating and cooling (thermal cycles) frequencies experienced during these time intervals.

The microstructures observed at the L20 section from different builds were found to be significantly different from each other, as can be seen from Figure 5a. In the NTI sample, the microstructure is primarily very disordered dendritic structures with the large globules of the δ and Laves phases mixed in randomly. In the LTI specimen, dendritic structures become more ordered, the δ structure and Lave phase become more dispersed in an interdendritic manner, and some equiaxed structures appear. In the HTI sample, the microstructure appears as an even mixture of very fine dendritic and equiaxed structures in which the δ and Laves phases are very well dispersed. Compared with the LTI specimen, more equiaxed structures were observed in the HTI sample. 

Moving up to the L50 section, the exhibited microstructures changed significantly from those of the lower sections (i.e., L20), as can be seen from Figure 4b. For the NTI sample, its microstructure changes from disordered dendritic structures to primarily equiaxed structures with the γ matrix becoming clearly visible. The large clumps of the δ and Laves phases are still mixed in randomly. In the LTI sample, the microstructure continues to show a mixture of equiaxed and dendritic structures. However, the δ and Laves structures disperse in both intergranular and interdendritic ways, forming very wide grain boundaries. In the HTI sample, long, fine dendritic structures have formed with both smaller dendritic and equiaxed structures dispersed throughout, while the δ and Laves phases are very well dispersed. 

After heat treatment, the microstructures appear to have changed into the expected matrices of γ, γ′, and γ″, with many residual structures from the original microstructure showing through. In the NTI sample, the bulk microstructure was rendered in an equiaxed manner, inside which the γ matrix is clearly visible. Some of the δ phases dispersed into the matrix to form much thicker grain boundaries; however, much of the δ and Laves structures still remained clumped randomly. In the LTI sample, the equiaxed structures were primarily developed during the heat treatment, meanwhile the previously dendritic structures were developed into the γ matrix. Some of the δ and Laves phases dispersed into intergranular regions or dissolved; however, much of them remained in what were the interdendritic regions. In the HTI sample, fine equiaxed structures were developed and filled with the γ matrix. Such morphology seems very similar to the fine dendritic structures before the heat treatment. Similar to the NTI and LTI samples, in the HTI sample, the Laves and δ phases either dispersed in an intergranular fashion or remained in the interdendritic regions, but the quantities of those phases were much smaller compared to the other two samples, as can be seen from Figure 5c. 

It was observed from Figure 5 that the NTI build had the highest amount of δ and Laves phases, followed by the LTI and HTI builds. The δ and Laves phases are caused by the segregation of refractory elements at highly elevated temperatures. This segregation would have been most prevalent at initial deposition, where the melt pool temperatures were significantly superheated. Furthermore, the difference between the quantities of those phases observed in the LTI and HTI samples is due to the decrease in the maximum melt pool temperature for layers above the SAZ: the higher-numbered layers dominated by environmental cooling. Meanwhile, the introduced inter-layer time intervals, which generally increased cooling rates, allowed for cooling to occur at slightly lower temperatures and the subsequent depositions were no longer significant enough to drive the microstructural changes. These cooling rates and the temperatures achieved after introducing the inter-layer time intervals yielded a re-homogenization effect to counteract the atomic segregation. 

The microstructural analysis results revealed a mixture of equiaxed and dendritic structures near the edge of the samples, which changes to entirely dendritic structures as the location moves away from the circumference towards the center of the samples. The differences between the microstructures at the circumference of the builds and those close to the center section of the builds are attributed to the different thermal histories over the surface and inside the samples. Those different thermal histories are caused by the difference between the surface temperature and the interior temperature, which is a result of different heat transfer modes. On the surface, the dominating heat transfer modes include convection and radiation, while inside the specimen, the heat transfer mode becomes more conduction dominant.

Thermal histories measured during the DED-L process show that for the LTI build, the surface temperature exceeded the annealing temperature of 924 °C for about 3.5 min at L20 and over 4 min at L40. Even though those times are not long enough to replicate the effects of the solution annealing treatment (which usually takes at least half an hour), the extension of the thermal history due to the introduction of the time interval did cause further changes in the microstructure and the dispersal of clumps of the Laves phase formed in the NTI build. The standard heat treatment schedule for wrought Inconel 718 [16] suggests that the solution treatment should be conducted at 924 °C for half an hour before dual aging. However, the microstructural analysis results show that due to the rapid cooling associated with the DED-L process, the detrimental δ and Laves phases could not be completely dispersed during the treatment and still presented themselves in the final microstructure. The appearance of the δ and Laves phases in the heat-treated samples suggests that the temperature and length of the solution treatment were not sufficient to dissolve these phases, and therefore there was not enough Ni for the full formation of the γ′ and γ″ precipitates. However, it appears that a higher inter-layer time interval (i.e., 10 s) can help to dissolve the δ and Laves phases. This suggests that the current standard schedules for solution treatment should be modified for DED-L Inconel 718 by increasing either the solution annealing temperature and/or the time to achieve a full homogenization. 

## 4. Conclusions

This study has investigated the effects of inter-layer time intervals and post-process heat treatment on microstructural properties of Inconel 718 fabricated via the powder-fed DED-L process. Dual wavelength pyrometry combined with infrared (IR) thermography was used to measure melt pool temperature distributions and heat-affected zone (HAZ) temperatures, respectively. The results demonstrate that increasing DED-L layer-to-layer time intervals: (1) reduces melt pool peak temperatures and agitation, (2) generates higher cooling rates and more uniform HAZ temperature distributions, and (3) promotes finer microstructures with reduced detrimental phases, even though heat treatment alone proves insufficient for their complete elimination. These findings suggest the significant potential of tailoring DED-L processes via time interval optimization to achieve the desired microstructural outcomes. Detailed conclusions are provided below.

1. The maximum melt pool temperature increases with layer number, peaks, and then decreases and levels out, with the acuteness of this trend decreasing as the DED-L inter-layer time interval increases. This phenomenon is attributed to the maximum melt pool temperature being sensitive to the part’s conduction and convection/radiation (environmental) thermal resistance. As the build height increases, surface area and volume increase, and the thermal resistance due to convection/radiation decreases to allow for more thermal energy stored in the part to dissipate. The layers below this critical point are more ‘substrate affected’ (conduction dominant), thus defining a substrate-affected zone (SAZ), while layers above this point are more convection-/radiation-affected due to the part having sufficient surface area to dissipate residual heat from the HAZ.
2. More ‘stable’ melt pools with less spatter generation exist when depositing on layers with less retained heat (and are at a lower initial temperature). Thus, longer time intervals imposed between layer deposits will result in cooler, less agitated melt pools.
3. The maximum melt pool temperature either decreases or increases during the deposition of a single layer. For layers being built within the SAZ, the maximum melt pool temperature generally increases with layer deposition time. With layers further from the SAZ, opposite trends can be observed.
4. Longer inter-layer time intervals lead to longer cooling times for the part being manufactured via DED-L. As a result, the melt pool temperature can achieve higher maximum temperatures during the initial scanning of a new layer. Higher cooling rates occur more often during DED-L processes with longer inter-layer time intervals.
5. The HAZ temperature cycled with each new layer deposit. Individual layer temperature responses became near-independent of layer number as the layer-to-layer time interval increased. This suggests that one can achieve more homogeneous microstructures in various-sized parts by employing longer inter-layer idles times.
6. Microstructures were found to contain large amounts of detrimental δ and Laves phases. Introducing layer-to-layer time intervals helped to dissolve some of these phases and disperse large clumps of them into the intergranular spaces. Microstructural evolutions were observed from the transition section close to the substrate to the middle section of the build. Heat treatment did not completely disperse these phases.

In summary, inter-layer time intervals during DED-L control microstructure similarity for scaled parts. Long time interval building strategies ensure similar microstructures in large/numerous DED-L Inconel 718 parts, addressing property performance for scaling to application-worthy components. Optimizing DED-L with time intervals and thermal monitoring can replace solution heat treatment for complex parts, reducing costs and times. IR imaging during DED-L offers ‘local’ process–structure–property–performance (PSPP) relationships, linking thermal phenomena with the post-AM microstructure, properties, and more. This empowers machine learning for real-time defect and/or microstructure detection in AM.

## Figures and Tables

**Table 1:** Process parameters utilized for the blown-powder DED-L of the investigated Inconel 718 samples.

Process Parameter	Value	Units
Laser power	350	W
Travel speed	8.5	mm/s
Powder flow rate	4.6	g/min
Hatch distance	529	μm
Hatch rotation	90	degrees
Layer thickness	760	μm

## Data Availability

Data are contained within the article.
